# Early resilience and epigenetic ageing: Results from the prospective Young Finns Study with a 31‐year follow‐up

**DOI:** 10.1111/acel.14394

**Published:** 2024-10-25

**Authors:** Aino Saarinen, Saara Marttila, Pashupati P. Mishra, Leo‐Pekka Lyytikäinen, Binisha Hamal Mishra, Emma Raitoharju, Nina Mononen, Mika Kähönen, Olli Raitakari, Terho Lehtimäki, Liisa Keltikangas‐Järvinen

**Affiliations:** ^1^ Department of Psychology and Logopedics, Faculty of Medicine University of Helsinki Helsinki Finland; ^2^ Department of Molecular Epidemiology, Cardiovascular Research Center Tampere, Faculty of Medicine and Health Technology Tampere University Tampere Finland; ^3^ Gerontology Research Center Tampere University Tampere Finland; ^4^ Department of Clinical Chemistry, Cardiovascular Research Center Tampere, Faculty of Medicine and Health Technology Tampere University Tampere Finland; ^5^ Department of Clinical Chemistry Fimlab Laboratories Tampere Finland; ^6^ Tampere University Hospital Wellbeing Services County of Pirkanmaa Tampere Finland; ^7^ Department of Clinical Physiology, Tampere University Hospital and Faculty of Medicine and Health Technology Tampere University Tampere Finland; ^8^ Research Centre of Applied and Preventive Cardiovascular Medicine University of Turku Turku Finland; ^9^ Centre for Population Health Research University of Turku and Turku University Hospital Turku Finland; ^10^ Department of Clinical Physiology and Nuclear Medicine Turku University Hospital Turku Finland

**Keywords:** ageing, biological clock, epigenetic ageing, epigenetic clock, resilience

## Abstract

Evidence is accumulating on the connection of early adversities and harsh family environment with epigenetic ageing. We investigated whether early psychosocial resilience is associated with epigenetic ageing in adulthood. We used the population‐based Young Finns data (*n* = 1593). Early psychosocial resilience was assessed in 1980–1989 across five broad domains: (1) index of psychological strength (self‐esteem at home/in general/at school, perceived possibilities to influence at home, internal life control), (2) index of social satisfaction (perceived support from family/friends and life satisfaction), (3) index of leisure time activities (hobbies and physical fitness), (4) index of responsible health behaviors (infrequent smoking or alcohol consumption), and (5) index of school career (school grades and adaptation). Epigenetic ages were calculated for blood samples from 2011, and the analyses were performed with variables describing age deviation (AgeDev_Hannum_, AgeDev_Horvath_, AgeDev_Pheno_, AgeDev_Grim_) and DunedinPACE. Covariates included early family environment, polygenic risk scores for schizophrenia and major depression, adulthood education, and adulthood health behaviors. All of the early resilience indexes were associated with lower levels of epigenetic ageing in adulthood, most consistently with AgeDev_Grim_ and DunedinPACE. The associations of psychological strength and social satisfaction, in particular, seemed to be non‐linear. In a smaller subsample (*n* = 289), high early resilience was related to lower AgeDev_Grim_ over a 25‐year follow‐up in those who had high “baseline” levels of AgeDev_Grim_. In conclusion, early resilience seems to associate with lower level of epigenetic ageing in adulthood. Our results tentatively suggest that early resilience may increase “equality in epigenetic ageing” in a general population.

## Graphical abstract

Various domains of early psychosocial resilience were associated with lower epigenetic ageing in the population‐based sample of the Young Finns Study. Our results tentatively suggest that early resilience may increase “equality in epigenetic ageing” in a general population.

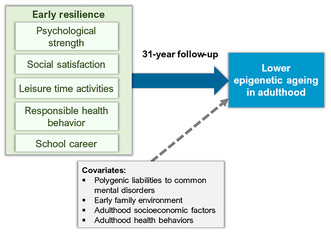

**Abbreviations**
BMI: body‐mass index
FDR: false discovery rate
YFS: the young finns study

## 1 INTRODUCTION

Epigenetic ageing can be assessed with three generations of epigenetic clocks. The first‐generation clocks include Hannum's and Horvath's clock that regress DNA methylation at distinct CpG sites on chronological age (Hannum et al., 2013; Horvath, 2013). The second‐generation clocks such as GrimAge and PhenoAge, in turn, incorporated also health biomarkers and phenotypes in their epigenetic age predictions (Levine et al., 2018; Lu et al., 2019). Also, a third‐generation clock has been emerged, that is, DunedinPACE, that was developed on the basis of within‐individual decline in 19 indicators of organ‐system integrity (e.g., BMI, leukocyte telomere length, HDL cholesterol) (Belsky et al., 2022).

Accelerated epigenetic ageing is related to a higher risk of somatic diseases such as cardiovascular diseases, cancer, reduced lung function, and diabetes (Oblak et al., 2021). A five‐year increase in Horvath and Hannum DNA methylation age correlates with an 8%–15% increased risk of mortality (Fransquet et al., 2019). Increased attention has been paid to identifying early‐life factors contributing to epigenetic ageing. An array of childhood adversities such as parental divorce, financial hardship, loss of a friend, sexual/emotional abuse, exposure to threats, violent victimization, and natural disasters, are related to epigenetic ageing in both childhood and adulthood (Copeland et al., 2022; Hamlat et al., 2021; Joshi et al., 2023; Jovanovic et al., 2017; Marini et al., 2020; McCrory et al., 2022; Simons et al., 2022; Sumner et al., 2019).

While the role of early adversities, stress exposures, and harsh environments on epigenetic ageing has become evident, the role of early resilience factors remains unclear. Resilience refers to a set of individual's internal strengths or external resources that promote achieving positive outcomes despite risky or challenging life conditions (Christmas & Khanlou, 2019; Zolkoski & Bullock, 2012). Accumulating evidence from previous literature identified resilience to include psychosocial factors such as self‐esteem, self‐efficacy, life satisfaction, social support, peer connectedness, cohesive family identity, coping strategies, sport involvement, educational achievements, and extracurricular activities (Christmas & Khanlou, 2019; Dumont & Provost, 1999; Martínez‐Martí & Ruch, 2017; Zolkoski & Bullock, 2012). Such resilience factors have been previously shown to predict, for example, lower incidence of cardiovascular outcomes (Park et al., 2022), better quality of life in people with somatic or mental illnesses (Chuang et al., 2023; Wu et al., 2015), and greater recovery after traumatic experiences (Iacoviello & Charney, 2014).

Regarding epigenetic ageing, to date, there are single studies reporting that psychological resilience, in terms of stress coping ability or social support, is related to decelerated GrimAge, PhenoAge and/or DunedinPACE in middle‐aged or elderly individuals (Bergquist et al., 2022; Hillmann et al., 2023; Rentscher et al., 2023). Additionally, psychological resilience, in terms of emotion regulation and self‐control, has been found to protect against the effects of stress on GrimAge in adults (Harvanek et al., 2021). To the best of our knowledge, however, evidence is lacking on resilience in childhood or adolescence in relation to epigenetic ageing.

We examined whether early psychosocial resilience is associated with epigenetic ageing in adulthood over a 31‐year follow‐up. The participants came from the prospective and population‐based Young Finns Study. Indicators of epigenetic age included in the study were the Horvath clock (Rentscher et al., 2023), Hannum clock (Hillmann et al., 2023), PhenoAge (Harvanek et al., 2021), and GrimAge (Raitakari et al., 2008). We utilized the measure of epigenetic age deviation, which is defined as the residual that results from regressing epigenetic age on chronological age (Marttila et al., 2021). These are denoted as AgeDev_Horvath_, AgeDev_Hannum_, AgeDev_Pheno_, and AgeDev_Grim_. In addition, we included a measure for pace of ageing, DunedinPACE (Chen et al., 2016). We ran sensitivity analyses in a small subsample who had data available on epigenetic age deviation in 1986. We first selected all relevant resilience‐related factors available in our dataset (identified as resilience factors in previous studies) and, then, using a factor‐analytical approach, we combined the single resilience factors into five broader resilience indexes: (Hannum et al., 2013) index of psychological strength, (Horvath, 2013) index of social satisfaction, (Levine et al., 2018) index of leisure time activities, (Lu et al., 2019) index of responsible health behaviors, and (Belsky et al., 2022) index of school career. Simultaneously, we took into consideration an array of background factors such as quality of family environment, polygenic liabilities for common mental disorders, and adulthood education and health behaviors.

## 2 MATERIALS AND METHODS

### 2.1 Participants

The Young Finns Study (YFS) is an on‐going prospective study that has started in 1980 (baseline assessment). Follow‐ups have been conducted in 1983, 1986, 1989, 1992, 1997, 2001, 2007, 2011/2012, and 2018–2020. Originally, a total of 4320 participants were invited (born in 1962, 1965, 1968, 1971, 1974, or 1977), and 3596 of them participated in the baseline study. In practice, the sampling was designed to include a population‐based sample of non‐institutionalized Finnish children, representative with regard to most crucial sociodemographic factors. In practice, the sampling was conducted in collaboration of five Finnish universities with medical schools (i.e., Universities of Helsinki, Turku, Tampere, Oulu, and Kuopio). A more detailed description of the YFS can be found elsewhere (Raitakari et al., 2008).

The Declaration of Helsinki has been followed throughout the study. The study design has been approved by the ethical committees of all the Finnish universities conducting the study. All the participants or their parents (participants aged <18 years) provided informed consent before participation.

**FIGURE 1:**
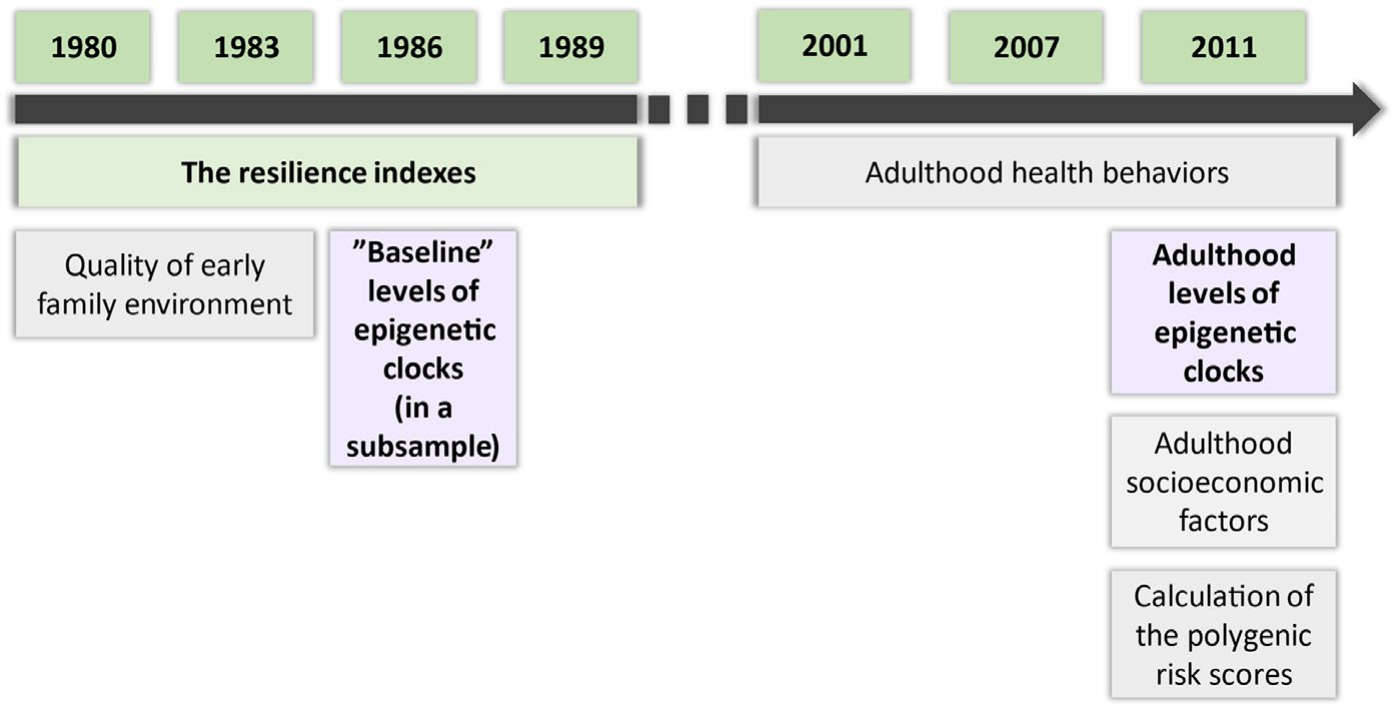
A summary of the study design.

In the main analyses, we included those participants who had successful DNA methylation profiling performed in 2011 and data available on early psychosocial resilience (1980, 1983, 1986, and/or 1989). We also added covariates in a stepwise method, including early family environment (1980/1983), polygenic risk scores for schizophrenia and major depression, and educational level and health behaviors in adulthood (2001/2007/2011). Accordingly, sample size varied between 855 and 1593 in the main analyses. The study design is illustrated in Figure 1.

### 2.2 Measures

#### 2.2.1 Indicators of epigenetic ageing

Epigenetic ages were calculated for blood samples taken in 2011. Genome‐wide DNA methylation levels from whole blood were obtained with Illumina Infinium HumanMethylation450 BeadChip (*n* = 180) or Illumina Infinium MethylationEPIC BeadChip (*n* = 1413) following standard protocol by Illumina. The data from each participant was processed using only one array (either 450 K or EPIC, without overlap). Preprocessing and normalization of the methylation data have been described in detail in (Marttila et al., 2021).

Indicators of epigenetic age included in the study were the Horvath clock (Horvath, 2013), Hannum clock (Hannum et al., 2013), PhenoAge (Levine et al., 2018), GrimAge (Lu et al., 2019). Here, we utilized the measure of epigenetic age deviation, which is defined as the residual that results from regressing epigenetic age on chronological age (Chen et al., 2016). These are denoted as AgeDev_Horvath_, AgeDev_Hannum_, AgeDev_Pheno_, and AgeDev_Grim_. In addition, we included a measure for pace of ageing, DunedinPACE (Belsky et al., 2022). Finally, in sensitivity analyses, we also utilized the derivatives of the Horvath and Hannum clocks, namely IEAA_Horvath_, IEAA_Hannum_, and EEAA_Hannum_ (Chen et al., 2016). Additionally, we used the principal component variables of the epigenetic clocks, including AgeDevPC_Horvath_, AgeDevPC_Hannum_, AgeDevPC_Pheno_, and AgeDevPC_Grim_ (Higgins‐Chen et al., 2022). All measures of epigenetic age deviation/acceleration were calculated according to published methods described above.

Also, we ran sensitivity analyses in a small subsample who had data available on epigenetic age deviation in 1986 (*n* = 289). In those analyses, we used AgeDev_Horvath_, AgeDev_Hannum_, AgeDev_Pheno_, and AgeDev_Grim_ (in 1986) that were calculated in similarly to the corresponding variables in adulthood (2011).

#### 2.2.2 Early psychosocial resilience

First, we began by reading a variety of previously published research papers to determine which kinds of factors have been included in the concept of resilience (Christmas & Khanlou, 2019; Dumont & Provost, 1999; Martínez‐Martí & Ruch, 2017; Zolkoski & Bullock, 2012). Next, we collected all relevant factors available in our dataset. Finally, we conducted a factor analysis to reduce the number of resilience‐related variables. The factor analyses resulted in five latent factors of early psychosocial resilience: (Hannum et al., 2013) index of psychological strength, (Horvath, 2013) index of social satisfaction, (Levine et al., 2018) index of leisure time activities, (Lu et al., 2019) index of responsible health behaviors, and (Belsky et al., 2022) index of school career. Also, we calculated a total score of early psychosocial resilience over all the domains. All the indexes were scaled so that higher scores referred to higher psychosocial resilience. The domains were self‐reported by the child/adolescent, except for the index of school career that was reported by parents. When calculating the indexes, we combined data from the baseline measurement (1980) and three follow‐ups (1983, 1986, 1989). The main contents of the indexes are described in Table 1. A more detailed description is provided in Supplementary Methods and Tables S1 and S2.

**TABLE 1:** Early psychosocial resilience.

Index of psychological strength	Index of social satisfaction	Index of leisure time activities	Index of responsible health behaviors	Index of school career
Self‐esteem across different life domains (home, school, in general) Perceived possibilities to influence at home Internal life control (low learned helplessness)	Perceived support from family Perceived support from friends Satisfaction with life/family	Frequency of physical activity (outside school lessons) Participation in sports club School grade for physical activity Number of hobbies Active participation in leisure time activities	Infrequent smoking Infrequent consumption of alcohol beverages (beer, wine, long‐drink, liquor)	Grade point average Lack of detention assigned to the child Lack of teacher's home reminders about the child's behavior No need for special support at school

#### 2.2.3 Covariates

Covariates included early family environment (1980/1983), polygenic risk scores for schizophrenia and major depression, adulthood educational level (2011), and adulthood health behaviors (daily smoking status, BMI, alcohol consumption, physical activity in 2001, 2007, and/or 2011). For further details, please see Supplementary Methods. We selected those covariates since also the previous studies on resilience and epigenetic ageing have controlled for adulthood health behaviors and education (Bergquist et al., 2022; Harvanek et al., 2021; Hillmann et al., 2023; Rentscher et al., 2023). Another reason for selecting these covariates was that smoking, alcohol consumption, obesity, or physical inactivity (Huang et al., 2019; Kresovich et al., 2021; Rosen et al., 2018), socioeconomic adversities (Fiorito et al., 2017; Oblak et al., 2021; Simons et al., 2016), early family adversities (Joshi et al., 2023), and severe psychiatric disorders such as schizophrenia or depression (Chrusciel et al., 2022; Han et al., 2018) are related to accelerated epigenetic ageing and, thus, may act as potential confounders.

### 2.3 Statistical analyses

Data was analyzed using Stata SE 14.0. First, we used regression analyses to examine whether early psychosocial resilience predicts indicators of epigenetic age deviation/acceleration. We estimated a separate model for each outcome: AgeDev_Hannum_, AgeDev_Horvath_, AgeDev_Pheno_, AgeDev_Grim_, and DunedinPACE. In the main analyses, we predicted the epigenetic outcomes, first, by the total score of early resilience and, then, separately by each single domain of early resilience. Analyses were run with two different sets of covariates. All the resilience indexes were approximately normally distributed, and we obtained no significant heteroscedasticity in the analyses. Models 1 were adjusted for sex, array type (450 K or EPIC), and smoking status (in 2011). Models 2 were additionally adjusted for early family risk polygenic risk scores for schizophrenia and major depression. Models 3 (full‐adjusted) were adjusted also for adulthood education and health behaviors (physical activity, alcohol consumption, BMI). Since previous studies have obtained non‐linear associations in resilience studies, we also examined possible polynomial associations of early resilience with indicators of epigenetic age deviation/acceleration. That is, we added index^2^ and index^3^ (i.e., the resilience index squared or cubed) as predictors in the model in case they were statistically significant. We also used false discovery rate (FDR) correction for multiple testing with Benjamini–Hochberg method. The method compares each individual *p*‐value to a critical value that is determined based on the desired false discovery rate control. In our study, this method was applied to all the *p*‐values reported in the tables.

## 3 RESULTS

### 3.1 Sample statistics

Descriptive statistics of the sample are shown in Table 2. The participants were on average 42 years old, and 56.0% of them were female. Most participants (74.0%) had an academic‐level education.

**TABLE 2:** Descriptive statistics of the study sample.

	Mean (SD)	Frequency (%)	Measurement range (min; max)
Age (2011)	42.14 (4.93)		34; 49
Sex (female)		892 (56.0)	
Early resilience			
Index of psychological strength	0.04 (0.99)		−3.66; 2.33
Index of social satisfaction	0.04 (0.96)		−3.32; 1.76
Index of leisure time activities	0.03 (0.98)		−3.05; 2.89
Index of responsible health behaviors	0.04 (0.97)		−8.54; 2.01
Index of school career	0.06 (0.99)		−4.25; 4.94
Total score of resilience	0.06 (0.94)		−4.20; 2.94
Childhood family risk	−0.10 (0.93)		−2.26; 4.00
Educational level			
Comprehensive school		30 (1.9)	
Occupational school or high school		379 (24.1)	
Academic level		1165 (74.0)	
Daily smoking status		224 (14.1)	
Alcohol consumption	0.84 (1.11)		0.00; 14.30
Physical activity	8.86 (1.60)		5.00; 14.67
BMI	25.98 (4.61)		17.06; 54.47
AgeDev_Horvath_	0.07 (4.18)		−22.69; 19.49
AgeDev_Hannum_	0.06 (4.16)		−10.22; 14.17
AgeDev_Pheno_	0.10 (5.34)		−17.47; 20.11
AgeDev_Grim_	−0.07 (3.66)		−9.21; 16.14
DunedinPACE	0.94 (0.10)		0.61; 1.35

*Note*: This table includes participants who were included in at least one analysis (*n* = 1593).

Next, we examined possible differences between included and dropped‐out participants with regard to the study variables. The full results can be found in Table S3. To summarize, women were more likely to participate than men. Additionally, included participants had slightly higher resilience scores in social satisfaction, responsible health behavior, and school career. There was no attrition bias in the epigenetic clock variables, except for AgeDevGrim where included participants had lower scores than dropped‐out participants.

### 3.2 Main analyses: Early resilience predicting epigenetic ageing over a 31‐year follow‐up

**FIGURE 2:**
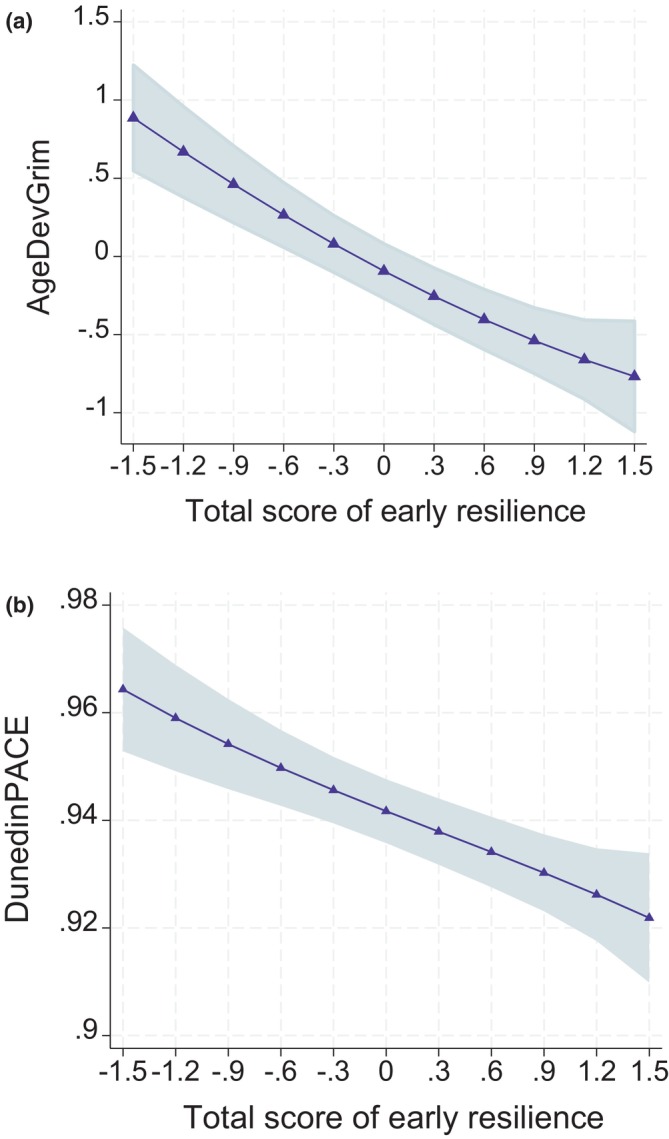
Model‐predicted means with 95% confidence intervals of (a) AgeDev_Grim_ and (b) DunedinPACE at different levels of total score of early resilience. Adjusted for sex, array type, and smoking status.

Table 3 presents the results when predicting epigenetic ageing in adulthood by the total score of early resilience. When adjusted for basic covariates (Models 1, adjusted for sex, smoking status, array type), high total scores of early resilience predicted lower levels of AgeDev_Pheno_ (B = −0.429, *p* = 0.005), AgeDev_Grim_ (B = −0.575, *p* = 2.29e‐12), and DunedinPACE (B = −0.015, *p* = 8.83e‐08) but not AgeDev_Horvath_ or AgeDev_Hannum_. After adjusting also for early family risk and polygenic risk scores for schizophrenia and major depression (Models 2), all these associations remained significant (the association between early resilience and AgeDev_Pheno_ did not sustain after FDR correction). In the fully‐adjusted models (adjusted also for adulthood education and health behaviors, Models 3), high total scores of early resilience predicted only lower levels of AgeDev_Grim_ (B = −0.311, *p* = 0.00078). The results are illustrated in Figure 2.

**TABLE 3:** Results of regression analyses when predicting epigenetic ageing by early resilience.

	Total score of early resilience Model 1 (*n* = 1593)			Total score of early resilience Model 2 (*n* = 1367)			Total score of early resilience Model 3 (*n* = 1346)		
	B	SE	*p*	B	SE	*p*	B	SE	*p*
AgeDev_Horvath_	−0.175	0.118	0.139	−0.179	0.134	0.181	−0.206	0.141	0.144
AgeDev_Hannum_	−0.065	0.117	0.578	−0.064	0.134	0.635	0.011	0.141	0.940
AgeDev_Pheno_	**−0.429**	**0.151**	**0.005** *	**−0.361**	**0.174**	**0.038**	−0.136	0.181	0.453
AgeDev_Grim_	**−0.575**	**0.081**	**2.29e‐12** *	**−0.485**	**0.090**	**8.34e‐08** *	**−0.311**	**0.092**	**0.00078** *
DunedinPACE	**−0.015**	**0.003**	**8.83e‐08** *	**−0.011**	**0.003**	**0.00043** *	−0.004	0.003	0.174

*Note*: Model 1: Adjusted for sex, array type, smoking status. Model 2: Additionally adjusted for early family risk and genetic risk for schizophrenia/depression. Model 3: Additionally adjusted also for adulthood health behaviors and adulthood educational level. There were no significant quadratic or cubic effects of the resilience score. Statistically significant (*p* < 0.05) associations are bolded.

* Statistical significance after FDR correction for multiple testing.

**FIGURE 3:**
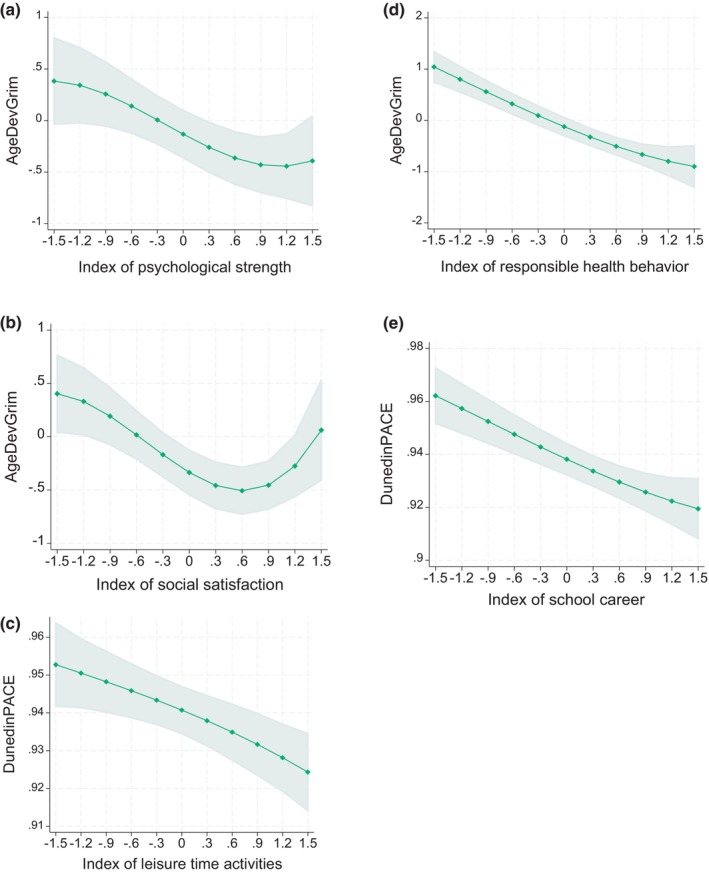
Model‐predicted means with 95% confidence intervals of AgeDev_Grim_ or DunedinPACE at different levels of (a) index of psychological strength, (b) index of social satisfaction, (c) leisure time activities, (d) index of responsible health behavior, or (e) index of school career. Adjusted for sex, array type, and smoking status.

Table 4 shows the results when predicting epigenetic ageing in adulthood separately by each domain of early resilience in Models 1 (adjusted for sex, array type, and smoking status). First, index of psychological strength was associated with AgeDev_Grim_ in a polynomial manner (see Figure 3a). Similarly, index of social satisfaction was associated with AgeDev_Grim_ in a polynomial manner (see Figure 3b). Third, index of leisure time activities had a negative linear association with AgeDev_Grim_ and DunedinPACE (see Figure 3c). Fourth, index of responsible health behaviors had a polynomial connection with AgeDev_Hannum_, AgeDev_Grim_, and DunedinPACE. This finding is illustrated in Figure 3d. Finally, index of school career was related to lower AgeDev_Horvath_, AgeDev_Pheno_, AgeDev_Grim_, and DunedinPACE in a linear manner (see Figure 3e). A majority of these associations sustained also after FDR correction for multiple testing (please see the asterisks in Table 4). All the other associations were non‐significant. To summarize, the indexes of early resilience had most significant associations with AgeDev_Grim_ and DunedinPACE but fewer associations with AgeDev_Horvath_, AgeDev_Hannum_, or AgeDev_Pheno_.

**TABLE 4:** Results of regression analyses when predicting epigenetic ageing separately by each domain of early resilience.

	Index of psychological strength (*n* = 990)			Index of social satisfaction (*n* = 1333)			Index of leisure time activities (*n* = 1455)			Index of responsible health behavior (*n* = 1551)			Index of school career (*n* = 1394)		
	B	SE	*p*	B	SE	*p*	B	SE	*p*	B	SE	*p*	B	SE	*p*
AgeDev_Horvath_															
Index	−0.151	0.134	0.258	0.025	0.122	0.836	−0.116	0.112	0.301	−0.143	0.114	0.210	**−0.243**	**0.114**	**0.034**
Index^2^	n.s.			n.s.			n.s.			n.s.			n.s.		
Index^3^	n.s.			n.s.			n.s.			n.s.			n.s.		
AgeDev_Hannum_															
Index	0.013	0.132	0.923	−0.040	0.121	0.738	0.082	0.111	0.463	0.016	0.129	0.903	−0.116	0.116	0.318
Index^2^	n.s.			n.s.			n.s.			0.191	0.099	0.054	n.s.		
Index^3^	n.s.			n.s.			n.s.			**0.029**	**0.014**	**0.041**	n.s.		
AgeDev_Pheno_															
Index	−0.299	0.174	0.086	0.004	0.157	0.979	−0.241	0.143	0.092	−0.260	0.147	0.076	**−0.378**	**0.149**	**0.011** *
Index^2^	n.s.			n.s.			n.s.			n.s.			n.s.		
Index^3^	n.s.			n.s.			n.s.			n.s.			n.s.		
AgeDev_Grim_															
Index	**−0.451**	**0.151**	**0.003** *	**−0.498**	**0.132**	**0.00018** *	**−0.161**	**0.077**	**0.038**	**−0.700**	**0.088**	**3.47e‐15** *	**−0.396**	**0.080**	**8.27e‐07** *
Index^2^	0.057	0.087	0.512	**0.057**	**0.087**	**0.0078** *	n.s.			0.086	0.067	0.201	n.s.		
Index^3^	**0.086**	**0.043**	**0.045**	**0.086**	**0.043**	**0.00039** *	n.s.			**0.023**	**0.009**	**0.015** *	n.s.		
DunedinPACE															
Index	−0.001	0.003	0.705	−0.001	0.003	0.752	**−0.010**	**0.003**	**0.00022** *	**−0.007**	**0.003**	**0.024**	**−0.014**	**0.003**	**1.31e‐07** *
Index^2^	n.s.			n.s.			n.s.			**0.008**	**0.002**	**0.001** *	n.s.		
Index^3^	n.s.			n.s.			n.s.			**0.001**	**0.000**	**0.001** *	n.s.		

*Note*: Adjusted for sex, smoking status, and array type. Index^2^ and Index^3^ refer to the quadratic or cubed effect of the index, respectively. We added Index^2^ and/or Index^3^ to each model in case they were statistically significant. Statistically significant (*p* < 0.05) associations are bolded.

* Statistical significance after FDR correction for multiple testing.

The results of Models 2 (adjusted also for early family environment and polygenic risk scores for schizophrenia and major depression) are presented in Table S4. Briefly, most results were replicated. The most notable change was that the index of psychological strength did not have a significant association with any measure of epigenetic ageing. The other resilience indexes still had associations with at least one measure of epigenetic ageing (i.e., AgeDev_Grim_, DunedinPACE, AgeDev_Pheno_, and/or AgeDev_Hannum_), also after FDR correction for multiple testing.

The results of Models 3 (adjusted also for adulthood education and health behaviors) can be found in Table S5. Now, the association of index of leisure time activities with epigenetic ageing disappeared. The index of social satisfaction, index of responsible health behavior, and index of school career were still associated with AgeDev_Grim_, DunedinPACE, and/or AgeDev_Hannum_ in these full‐adjusted models (also after FDR correction for multiple testing).

### 3.3 Sensitivity analyses

#### 3.3.1 Analyses with array type

As a minority of the data set were analysed with 450 K array, we reran the interaction analyses so that only EPIC array data were included. The results are presented in Table S6. To summarize, all the results of the main analyses were replicated. Besides, two additional associations emerged: the index of psychological strength had a linear association with AgeDev_Pheno_, and the index of leisure time activities was associated with AgeDev_Horvath_. After FDR correction, all the indexes had significant associations with at least one epigenetic clock, except for the associations between psychological strength and epigenetic clocks that were non‐significant after FDR correction.

#### 3.3.2 Analyses accounting for cell type composition

Then, we reran the main analyses so that cell types were controlled for (CD8^+^ T cells, CD4^+^ T cells, NK cells, B cells, and monocytes, proportions estimated from DNA methylation data). To avoid the risk of over‐fitting, we excluded one cell type from the covariates (i.e., granulocytes). Blood cell proportions were estimated with the Houseman method (Houseman et al., 2012) using the minfi R package. The results can be found in Table S7. To summarize, the results were mostly replicated. After FDR correction for multiple testing, the same associations between the resilience indexes and epigenetic clocks were statistically significant in the main analyses versus these sensitivity analyses, except for three associations: the index of responsible health behavior started to predict AgeDev_Pheno_ (*p* = 0.006), the index of leisure time activities started to predict AgeDev_Hannum_ (*p* = 0.007), and the association between the index of psychological strength and AgeDev_Grim_ did not sustain after FDR correction (*p* = 0.025).

#### 3.3.3 Analyses with derivatives of the clocks

Next, as a sensitivity analysis, we used the derivatives of the epigenetic clocks, namely, IEAA_Horvath_, IEAA_Hannum_, EEAA_Hannum_, AgeDevPC_Horvath_, AgeDevPC_Hannum_, AgeDevPC_Pheno_, and AgeDevPC_Grim_. The results can be found in Table S8. When comparing the results between AgeDev_Horvath_ and IEAA_Horvath_/AgeDevPC_Horvath_, the results were replicated. When comparing the results between AgeDev_Pheno_ and AgeDevPC_Pheno_, the association with the index of school career was replicated. When comparing the results between AgeDev_Grim_ and AgeDevPC_Grim_, all the results were replicated with one exception (a non‐significant association of index of social satisfaction and AgeDevPC_Grim_). Finally, regarding the Hannum clocks, we found that the index of leisure time activities had stronger associations with IEAA_Hannum_ and EEAA_Hannum_ than AgeDev_Hannum_, while the index of responsible health behavior had stronger associations with AgeDev_Hannum_ than with its derivatives.

#### 3.3.4 Longitudinal analyses in a subsample

**FIGURE 4:**
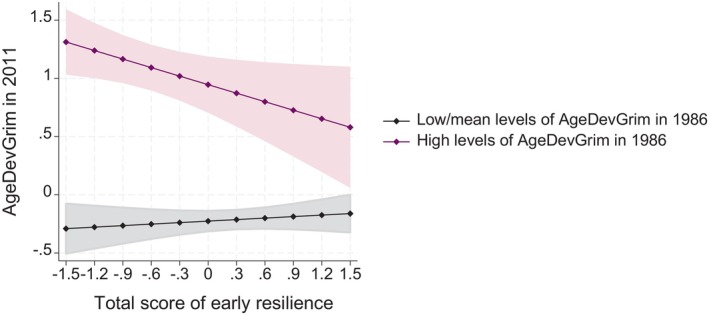
Model‐predicted means with 95% confidence intervals of AgeDev_Grim_ in 2011 at different levels of total score of early resilience and separately for participants with high levels (+1 SD) and low/mean levels of AgeDev_Grim_ in 1986. Adjusted for sex, array type, and smoking status.

Finally, we conducted longitudinal analyses in a subsample of participants who had data available on epigenetic ageing in 1986 (*n* = 289). Here, we were particularly interested whether early resilience could protect against accelerated epigenetic ageing in adulthood especially those individuals with high levels of “baseline” epigenetic ageing. Thus, we examined the moderating effect of early resilience on the association between epigenetic clocks between 1986 and 2011 (AgeDev_Horvath_, AgeDev_Hannum_, AgeDev_Pheno_, AgeDev_Grim_). The results are presented in Table S9. To summarize, we found an interaction between early resilience and AgeDev_Grim_ in 1986 when predicting AgeDev_Grim_ in 2011 (*p* = 0.047), indicating that higher total scores of early resilience predicted lower levels of AgeDev_Grim_ in 2011 in those who had high levels of AgeDev_Grim_ in 1986 (see Figure 4). This association, however, did not sustain after FDR correction for multiple testing. When examining the other indicators of epigenetic ageing (AgeDev_Horvath_, AgeDev_Hannum_, AgeDev_Pheno_, DunedinPACE), we did not find any significant interactions.

Finally, we also examined the main effects of early resilience and “baseline” epigenetic clocks without the interaction effect (i.e., assuming that early resilience could have a similar effect on adulthood epigenetic ageing independently of the “baseline” level of epigenetic clock). There were not any significant main effects in these analyses (*p* = 0.545–0.938).

## 4 DISCUSSION

To the best of our knowledge, this was the first study to investigate the role of early resilience on epigenetic ageing in adulthood over decades of follow‐up. Across a broad range of early resilience indexes, including psychological strength, social satisfaction, school career, responsible health behaviors, and leisure time activities, we found associations with lower levels of epigenetic ageing in adulthood. Many of those associations persisted even after accounting for the quality of early family environment, polygenic risk scores for common mental disorders, and adulthood education and health behaviors. Additionally, in smaller subsample who had data available on “baseline” epigenetic age in 1986 (*n* = 289), we found that high early resilience longitudinally predicted lower levels of AgeDev_Grim_ over a 25‐year follow‐up in those with high “baseline” levels of AgeDev_Grim_.

The associations were most consistent with AgeDev_Grim_ and DunedinPACE. This is in accordance with, first, previous resilience studies in older age groups, where psychological resilience in middle age or older has been cross‐sectionally linked to lower levels of AgeDev_Grim_ or DunedinPACE (Bergquist et al., 2022; Harvanek et al., 2021; Hillmann et al., 2023; Rentscher et al., 2023). Second, our results are in accordance with the evidence that AgeDev_Grim_ and DunedinPACE have been built at least partly utilizing adulthood metabolite levels and are among the strongest predictors of mortality and ageing‐related clinical conditions (Belsky et al., 2022; Lu et al., 2019; McCrory et al., 2021).

High early resilience was longitudinally related to a lower adulthood level of AgeDev_Grim_ in those with high scores of “baseline” epigenetic age, while in those with low or moderate “baseline” levels of AgeDev_Grim_, however, high early resilience did not appear to have such a protective effect against epigenetic ageing in later life. Therefore, our results suggest that high early resilience may be related to lesser variation in middle‐age epigenetic ageing and, thus, to increase “equality in epigenetic ageing” in a general population.

While the index of school career had linear associations with epigenetic age (i.e., “more is better”), the indexes of psychological strength and social satisfaction appeared to have a rather non‐linear association with epigenetic ageing. Thus, very high levels of psychological strength or social satisfaction may not have an additive protective effect against epigenetic ageing. Although possibly non‐intuitive at first sight, this is not a completely new finding. It has been reported that high resilience is associated with higher epigenetic ageing in soldiers with posttraumatic stress disorder (Mehta et al., 2018) and that high self‐control is related to higher levels of Hannum/Horvath clocks among rural African‐American youths living in disadvantaged environments (Brody et al., 2016). Thus, extreme high levels of psychological resilience may possibly have “biological costs” or to be “only skin deep” if living in harsh environments (Brody et al., 2016; Mehta et al., 2018).

Regarding limitations, the subsample of our longitudinal analyses of epigenetic ageing was limited (*n* = 289). Despite the results being in accordance with the main analyses (i.e., associations were found with the same epigenetic clock, namely AgeDev_Grim_) and thus plausible, those results must be treated with caution and replicated in larger samples. Second, we did not have follow‐up data on the resilience indexes in adulthood or middle age. Thus, the data did not allow us to investigate whether the early resilience factors are related to epigenetic ageing after accounting for similar resilience factors in adulthood.

In conclusion, our results from a general population sample provide evidence that early psychosocial resilience is longitudinally associated with epigenetic ageing over a 25‐year follow‐up period. The results underscore the importance of early favorable factors in epigenetic clocks such as AgeDev_Grim_ and DunedinPACE. Furthermore, our study indicates that these associations may be somewhat attenuated but not fully explained by confounders such as the quality of early family environment, polygenic liabilities for common mental disorders, or socioeconomic and health behavioral factors in adulthood.

## AUTHOR CONTRIBUTIONS

O.R., T.L., L.K‐J., and M.K. contributed to data collection. S.M., P.P.M., L.‐P.L., N.M., and E.R. contributed to data preprocessing. A.S. conducted the statistical analyses and wrote an initial draft. All authors contributed to commenting and writing of the manuscript.

## CONFLICT OF INTEREST STATEMENT

Authors declare no competing financial interests in relation to the work described.

## Supporting information


Data S1.

## Data Availability

The Cardiovascular Risk in Young Finns (YFS) dataset comprises health‐related participant data, and their use is therefore restricted under the regulations on professional secrecy (Act on the Openness of Government Activities, 612/1999) and on sensitive personal data (Personal Data Act, 523/1999, implementing the EU data protection directive 95/46/EC). Due to these legal restrictions, the data from this study cannot be stored in public repositories or otherwise made publicly available. However, data access may be permitted on a case by case basis upon request. Data sharing outside the group is done in collaboration with YFS group and requires a data‐sharing agreement. Investigators can submit an expression of interest to the chairman of the publication committee (Prof. Mika Kähönen, Tampere University, Finland, mika.kahonen@tuni.fi).
