# Supplementary material for: Early resilience and epigenetic ageing: Results from the prospective Young Finns Study with a 31‐year follow‐up
Source: Aging Cell. 2024 Oct 25;24(2):e14394. doi: 10.1111/acel.14394 (PMC11822653; doi:10.1111/acel.14394)
Supplement: Supplementary file 1 — Data S1. [file ACEL-24-e14394-s001.docx]

**Supplementary Material**

**S1. Assessment of early psychosocial resilience**

First, we collected all available (1) psychological, (2) lifestyle-related, and (3) school-related factors of resilience that have been identified as resilience factors in the research literature. All the factors were scaled so that higher scores referred to higher psychosocial resilience. We used data from the baseline measurement (1980) and three follow-ups (1983, 1986, and/or 1989). The measurement years of each resilience factor are described in **Supplementary Table 1**.

**Supplementary Table 1.** Measurement years of each resilience factor.

|  | **Measurement year** | | | |
| --- | --- | --- | --- | --- |
| **Resilience factor** | **1980** | **1983** | **1986** | **1989** |
| Satisfaction with life/family |  |  |  | - (n = 1338) |
| Perceived support from family |  |  |  | - (n = 1351) |
| Perceived support from friends |  |  |  | - (n = 1350) |
| Perceived possibilities to influence at home |  |  | - (n = 1001) |  |
| Internal life control |  |  | - (n = 1002) |  |
| Self-esteem at home | - (n = 872) |  | - (n = 452) |  |
| Self-esteem in general / at school | - (n = 873) |  | - (n = 1002) |  |
| Self-esteem in social relationships | - (n = 871) |  | - (n = 452) |  |
| Leisure time activities |  | - (n = 951) | - (n = 1070) | - (n = 1368) |
| Expected physical fitness | - (n = 1162) | - (n = 1188) | - (n = 1281) | - (n = 1362) |
| Infrequent alcohol consumption | - (n = 859) | - (n = 930) | - (n = 879) | - (n = 1326) |
| Infrequent smoking | - (n = 850) | - (n = 928) | - (n = 1055) | - (n = 1358) |
| School performance | - (n = 1003) | - (n = 1089) | - (n = 571) | - (n = 389) |
| School adaptation | - (n = 1568) | - (n = 1378) | - (n = 661) |  |

**S1.1. Psychological domains of resilience**

**Experienced satisfaction with life/family** (in 1989) was assessed with a four-item questionnaire (“I am very happy with my current life”, “My family and me are not doing well at this point” [reversed], “I am happy with my school/work”, “I am very unhappy with my life and I do not think it will get better”). The items were responded with a five-point Likert-scale (1 = totally disagree, 5 = totally agree). We calculated a mean of the items for all the participants who had responded to at least 50 % of the items.

**Perceived support from family and friends** was assessed with the Multidimensional Scale of Perceived Social Support in 1989 (MSPSS) (Zimet, 1988) that is found to be a valid and reliable measure of perceived social support (Hardan-Khalil, 2015; Rajabi, 2012). Social support from family (Cronbach’s alpha = 0.91) was measured with four items (“I get real support from my family”, “My family supports me always when I need help”, “I can discuss my problems with my family”, “When making important decisions I get help from my family”). Social support from friends (Cronbach’s alpha = 0.95) was assessed with eight items (e.g., “I have a close friend who supports me when I need help”, “I have a close friend who I can share my joys and griefs with”, “I have a close friend who comforts me”, “I can discuss my problems with my friends”). All the items were responded with a five-point scale (1 = totally agree; 5 = totally disagree). We calculated a mean score of social support from family and a mean score of social support from friends for all the participants who had responded to at least 50 % of the items.

**Perceived possibilities to influence at home** and **internal life control** were assessed with the items derived from the Locus of Control Scale for Children in 1986 (LCSS) (Nowicki & Strickland, 1973). The questionnaire included a separate response scale for younger age cohorts (no/yes) and for older age cohorts (a five-point scale). After conducting an exploratory factor analysis of the LCSS scale, the results indicated two latent factors. The first factor (named as perceived possibilities to influence at home) included six items (e.g., “Do you feel that parents mostly listen what their children have to say”, “Do you feel that most of the time you only have a little say in what your family decides to do”, “Do you feel that your parents' opinion is almost impossible to change in any matter”). The second factor (named as internal life control, i.e., low learned helplessness) included seven items (e.g., “Do you believe that most problems are best solved by themselves if you can only manage not to interfere with them”, “Do you feel that one of the best ways to deal with most problems is to simply not think about them”, “Do you believe that when bad things are meant to happen, they just happen, regardless of what you try to do to prevent them”). We calculated a mean score of perceived possibilities to influence at home and a mean score of internal life control for all the participants who had responded to at least 50 % of the items.

**Self-esteem** was assessed in 1980 and 1986 with the Coopersmith Self-Esteem Inventory, including 20 items. In 1980, the items were responded with a three-point scale (1 = no, 2 = not sure, 3 = yes) and in 1986 with a five-point scale (1 = totally disagree, 5 = totally agree). In line with a previous study on this same dataset (Keltikangas-Järvinen, 1992), we formed three factors of the scale referring to self-esteem at home (six items, Cronbach’s alpha = 0.70, e.g., “My parents usually take into consideration my emotions”, “My parents and me usually have fun together”), self-esteem at school/in general (nine items, Cronbach’s alpha = 0.70, e.g., “I commonly lose my courage at school”, “I am a failed person”), and self-esteem in social relationships (five items, e.g., “I am popular with my peers”, “People usually like me”). For each measurement year, we calculated a mean score of self-esteem at home, self-esteem at school/in general, and self-esteem in social relationships for all the participants who had responded to at least 50 % of the items in at least one measurement year.

**S1.2. Lifestyle-related domains of resilience**

**Leisure activities.** Each child/adolescent reported his/her number of hobbies (in 1983, 1986, 1989) with a seven-point scale (1 = many hobbies, 7 = few hobbies). We reversed the scale and calculated a mean score of number of hobbies for all the participants who had responded to the question in at least one measurement year. Active participation in leisure time activities was reported in 1983, 1986, and 1989 with a five-point scale (1 = totally disagree, 5 = totally agree). We calculated a mean score of the variable for all the participants (over the measurement years) who had responded to the question in at least one measurement year.

**Expected physical fitness.** Frequency of physical activity (outside school lessons and at least 30 minutes per time) was assessed with a seven-point scale (1 = not at all, 7 = every day) in 1980, 1983, 1986, and 1989. We calculated a mean score between measurement years for all the participants who had responded to the question in at least one measurement year. Participation in sports club exercises (1980, 1983, 1986, 1989) was reported with a six-point Likert-scale (1 = many times and many hours in a week, 6 = not at all). We reversed the scale and calculated a mean score between measurement years for all the participants who had responded to the question in at least one measurement year. School grade for physical activity was reported in 1980, 1983, 1986, and 1989 with a scale from 4 (rejected) to 10 (excellent). We calculated a mean score of between the measurement years for all the participants who had responded to the question in at least one measurement year.

**Frequency of smoking** was reported in 1980, 1983, 1986, and 1989 with a five-point scale (1 = daily smoking, 5 = never smoked). We calculated a mean score of the responses for all the participants who had responded to the question in at least one measurement year.

**Consumption of alcohol beverages** (beer, wine, long-drink, liquor) was assessed in 1980, 1983, 1986, and 1989 with a self-report scale. In 1980 and 1983, the scale included three items (frequency of drinking beer, wine, or liquor) responded with a nine-point scale (1 = daily, 9 = never). In 1986 and 1989, the scale included five items (frequency of drinking beer, wine, long-drink or cider, liquor) responded with a five-point scale (1 = daily, 5 = never). For each year, we calculated a total score of the items for all the participants who had responded to the items. Finally, we calculated a total score of alcohol consumption over the measurement years for all the participants who had reported alcohol consumption in at least one measurement year.

**S1.3. School-related domains of resilience**

**School performance.** Grade-point average (GPA) was reported by a parent in 1980, 1983, 1986, and 1989 with a continuous scale (4 = rejected, 10 = excellent). We calculated a mean score of GPA over the follow-up for all those who had data available in at least one measurement year.

**School adaptation.** School detention was assessed in 1980, 1983, and 1986 by asking a parent whether the child had been assigned with detention at school (yes/no). We formed a dichotomous score describing whether the child/adolescent was reported to be given detention in at least one measurement year (0 = yes, 1 = no). Home reminders were assessed in 1980, 1983, and 1986 by asking a parent whether the teacher had sent parents at least one home reminder about the child’s behavior (yes/no). We formed a dichotomous score describing whether the parent had received home reminder in at least one measurement year (0 = yes, 1 = no). Special education at school was evaluated in 1980, 1983, and 1986 by asking parents whether their child had been provided with special support at school (yes/no). Again, we formed a dichotomous score describing whether the child had been given special support at school in at least one measurement year (0 = yes, 1 = no).

**S1.4. Combining single resilience factors into broader resilience indexes**

At this point, we had encoded a total of 14 single resilience scores (satisfaction with life/family, perceived support from family, perceived support from friends, possibilities to influence at home, internal life control, self-esteem at home, self-esteem in general/at school, self-esteem in social relationships, leisure time activities, physical fitness, alcohol consumption, smoking, school performance, and school adaptation). Each resilience score was standardized within the age cohort (mean = 0, SD = 1).

Next, we conducted an exploratory factor analysis of the 14 single resilience factors to reduce the number of dimensions. The factor loadings of the resilience scores (rotated with oblimin quartimin) indicated a five-factor solution. The number of factors was defined on the basis of eigenvalues. The results are summarized in **Supplementary Table 2**. Briefly, the factor analysis indicated a five-factor solution of resilience: (1) “Psychological strength” (including possibilities to influence at home, internal life control, self-esteem at home, and self-esteem in general/at school), (2) “Satisfaction and support” (including satisfaction with life/family, perceived support from family and friends), (3) Leisure time activities (including hobbies and physical fitness), (4) “Responsible health behavior” (including infrequent alcohol consumption and smoking), and (5) “School career” (including school performance and school adaptation). The score of self-esteem in social relationships did not load to any of the factors and, thus, was excluded from the final resilience indexes. Finally, calculated a total score of the single resilience scores within each index (e.g., a mean score of satisfaction with life/family and perceived support from family and friends to indicate index of psychological strength). These resilience indexes were used in the analyses.

**Supplementary Table 2.** Factor loadings of the single resilience factors (rotated with oblimin quartimin).

| **Index** | **Resilience factor** | **Factor 1** | **Factor 2** | **Factor 3** | **Factor 4** | **Factor 5** |
| --- | --- | --- | --- | --- | --- | --- |
| **Index of social satisfaction** | Satisfaction with life/family | 0.029 | **0.562** | 0.021 | 0.066 | 0.006 |
|  | Perceived support from family | 0.012 | **0.643** | 0.000 | 0.065 | -0.055 |
|  | Perceived support from friends | -0.010 | **0.534** | 0.015 | -0.131 | 0.096 |
| **Index of psychological strength** | Possibilities to influence at home | **0.555** | 0.058 | -0.011 | -0.065 | 0.022 |
|  | Internal life control | **0.528** | -0.016 | 0.017 | 0.024 | 0.048 |
|  | Self-esteem at home | **0.412** | 0.079 | -0.014 | 0.118 | 0.010 |
|  | Self-esteem in general / at school | **0.515** | 0.009 | 0.052 | 0.003 | 0.014 |
| **Index of leisure time activities** | Leisure time activities | 0.031 | 0.027 | **0.687** | 0.024 | -0.045 |
|  | Physical fitness | -0.032 | -0.017 | **0.700** | -0.018 | 0.040 |
| **Index of responsible health behavior** | Alcohol consumption | -0.011 | 0.052 | -0.045 | **0.600** | -0.034 |
|  | Smoking | 0.004 | 0.004 | 0.073 | **0.598** | 0.095 |
| **Index of school career** | School performance | 0.145 | -0.013 | 0.085 | 0.174 | **0.358** |
|  | School adaptation | -0.017 | 0.024 | -0.033 | 0.029 | **0.350** |
| - (excluded) | Self-esteem in social relationships | 0.068 | 0.096 | 0.084 | -0.147 | 0.015 |

**S2. Assessment of early family environment**

Early family environment was assessed with a total score over three domains: (1) unfavorable emotional family atmosphere, (2) stressful life events, (3) adverse socioeconomic family environment. The contents of each domain are illustrated in **Supplementary Table 3**. All the questionnaires related to childhood family environment were fulfilled by a parent (mostly mother) in 1980. In case there were missing values in 1980, they were imputed using data from the 1983 follow-up. Similar cumulative risk scores have been used also previously (42). The main contents of the scores are described in **Supplementary** **Table 3**.

**Cumulative risk score of socioeconomic family risk** included the following factors: parents’ occupational status (1 = upper-grade non-manual worker, 2 = lower-grade non-manual worker, 3 = manual worker manual worker), parents’ educational level (1 = academic level, 2 = high school or occupational school, 3 = comprehensive school), family income (1 = more than 100 000 Finnish mark, 8 = less than 20 000 Finnish mark), unstable employment situation (1 = at least one parent was unemployed or in a long-term sick leave, 0 = other employment situations), and over-crowded apartment (family size in relation to number of rooms at home). Each item was standardized by age cohort (M = 0, SD = 1 within each age cohort), and we calculated a mean score of the standardized items.

**Cumulative score of stressful life events** included the following factors: change of residence (yes/no), number of change of school (continuous response), parental divorce (whether parents living together or separated), mother’s or father’s death, mother’s or father’s hospitalization within the past 12 months (number of days in hospital, ranging from “1 = no days” to “5 = more than 30 days”), and child’s hospitalization due to sickness or accident (yes/no).

**Cumulative score of emotional family atmosphere** included the following factors: emotional distance between the child and parent, parental intolerance toward the child, strict discipline toward the child, parental life dissatisfaction, mother’s or father’s mental disorder (no/yes), and mother’s or father’s frequent alcohol intoxication (ranging from “1 = never” to “8 = daily”). Emotional distance between the parent and child was evaluated with a four-item questionnaire (e.g., “The child is emotionally important for me”, ”I can realize myself with the child”). The items were responded with a 5-point scale (e.g., 1 = little, 5 = much). Parental intolerance toward the child was evaluated with a three-item scale (“I get nervous when spending time with the child”, “The child is a burden in challenging situations”, “The child consumes my time too much”). The items were responded with a 5-point scale (1 = frequently, 5 = never). Strict discipline toward the child was measured with a three-item scale (“Disciplinary actions are often needed at home due to child’s aggressiveness”; “Disciplinary actions do not affect the child enough”; “Disciplinary actions are necessary in the rearing of the child”). The items measuring parenting have been used also previously (43, 44). Parental life satisfaction was assessed with a three-item questionnaire measuring parent’s satisfaction in three life sectors: as a parent, spouse, and employee. The items were responded with a 5-point scale (1 = satisfied, 5 = dissatisfied). This questionnaire has been adapted from the Operation Family Study questionnaire (45) and has been used also previously (46, 47).

A more detailed description of the cumulative risk scores is available elsewhere (48). In the present study, we used a total score of early family environment over the three domains.

**S3. Assessment of lifestyle covariates in adulthood**

**Educational level** (2011) was classified into three categories (1 = comprehensive school, i.e., the nine first school years; 2 = high school or occupational school; 3 = academic level).

**Smoking** was assessed in 2011 with self-report questionnaire in 2011 (1 = daily smoking; 6 = having never smoked). We calculated a dichotomized variable (0 = not daily smoking; 1 = daily smoking).

**Alcohol consumption** was assessed in 2001, 2007, and 2011 by asking the participants to report their consumption of 1/3 l cans or bottles of beer, glasses (12 cl) of wine, and 4 cl shots of liquor or strong alcohol within the past week. We calculated a sum score of different beverages consumed within the past week. A similar index of alcohol consumption has been used also previously (49). In the analyses, we used a mean score over the measurement years if data available in at least one measurement year.

**Physical activity** was assessed in 2007, 2007, and 2011 with five items: (i) “How much breathlessness and sweating do you experience when you engage in sport or physical activity?”; (ii) “How often do you engage in sport or physical activity so that you get out of breath and sweat?”; (iii) “How many hours per week do you usually engage in sport or physical activity so that you get out of breath and sweat?”; (iv) “How much time do you usually spend in one session of sport of physical activity?”; (v) “Do you participate in organized physical activity (e.g. in sport club)?”. We calculated a total score for each measurement year. The same index of physical activity has been used also previously (50). In the analyses, we used a mean score of physical activity over the measurement years if data available in at least one measurement year.

**Body-mass index** **(BMI)** was also measured in 2001, 2007, and 2011. In the analyses, we used a mean over the measurement points if data available in at least one measurement year.

**S4. Calculation of polygenic risk scores for schizophrenia and major depression**

**Polygenic risk score for major depression.** Genomic DNA was extracted from peripheral blood leukocytes using a commercially available kit and Qiagen BioRobot M48 Workstation according to the manufacturer's instructions (Qiagen, Hilden, Germany). Genotyping was done for 2556 samples using custom build Illumina Human 670 k BeadChip atWelcome Trust Sanger Institute. Genotypes were called using Illuminus clustering algorithm. Samples that failed Sanger genotyping pipeline QC criteria (i.e., duplicated samples, heterozygosity, low call rate, or Sequenom fingerprint discrepancy) were excluded from analysis. Similarly, samples with sex discrepancy, low genotyping call rate (< 0.95) and possible relatedness (pi-hat > 0.2) were excluded from the analysis. Short Nucleotide Polymorphisms (SNPs) were excluded based on Hardy-Weinberg equilibrium test (p ≤ 1e-06), failed missingness test (call rate < 0.95) and failed frequency test (minor allele frequency < 0.01). After quality control, 546,677 genotyped SNPs were available for further analysis. Genotype imputation was performed using Minimac3 (Das et al., 2016) and 1000 G phase3 reference set on Michigan Imputation Server. A total of 102 depression related SNPs identified using genomic data from >800,000 individuals (p-value < 5 × 10−8) by (Howard et al., 2019) were used for calculation of weighted genetic risk score (GRS) for depression using Plink software (Purcell et al., 2007).

**Polygenic risk score for schizophrenia** was calculated using PRS-CS method (Ge et al., 2019), which infers posterior SNP effect sizes under continuous shrinkage (CS) priors using GWAS summary statistics and an external LD reference panel. The latest available schizophrenia GWAS results (Trubetskoy et al., 2022) were used as SNP summary statistics and HapMap 3 EUR as an external LD reference (The International HapMAP 3 Consortium, 2011).

**References**

Nowicki, S., & Strickland, B. R. (1973). A locus of control scale for children. *Journal of consulting and clinical psychology*, *40*(1), 148.

Ge, T., Chen, C. Y., Ni, Y., Feng, Y. C. A., & Smoller, J. W. (2019). Polygenic prediction via Bayesian regression and continuous shrinkage priors. *Nature communications*, *10*(1), 1776.

Howard, D. M., Adams, M. J., Clarke, T. K., Hafferty, J. D., Gibson, J., Shirali, M., ... & McIntosh, A. M. (2019). Genome-wide meta-analysis of depression identifies 102 independent variants and highlights the importance of the prefrontal brain regions. *Nature Neuroscience*, *22*(3), 343-352.

International HapMap 3 Consortium. (2010). Integrating common and rare genetic variation in diverse human populations. *Nature*, *467*(7311), 52.

Keltikangas-Järvinen, L. (1992). Self-esteem as a predictor of future school achievement. *European Journal of Psychology of Education*, *7*, 123-130.

Zimet, G. D., Dahlem, N. W., Zimet, S. G., & Farley, G. K. (1988). The multidimensional scale of perceived social support. *Journal of Personality Assessment*, 52(1), 30-41.

Hardan-Khalil, K., & Mayo, A. M. (2015). Psychometric properties of the multidimensional scale of perceived social support. *Clinical Nurse Specialist*, *29*(5), 258-261.

Purcell, S., Neale, B., Todd-Brown, K., Thomas, L., Ferreira, M. A., Bender, D., ... & Sham, P. C. (2007). PLINK: a tool set for whole-genome association and population-based linkage analyses. *The American Journal of Human Genetics*, *81*(3), 559-575.

Rajabi, G. (2012). The study of psychometric properties of the Multidimensional Scale Perceived Social Support. *International Journal of Behavioral Sciences*, *5*(4), 357-364.

Trubetskoy, V., Pardiñas, A. F., Qi, T., Panagiotaropoulou, G., Awasthi, S., Bigdeli, T. B., ... & Lazzeroni, L. C. (2022). Mapping genomic loci implicates genes and synaptic biology in schizophrenia. *Nature*, *604*(7906), 502-508.

**Supplementary Table 3.** Differences between included and dropped-out participants in the main study variables. Note: n.s. = non-significant

|  | Mean difference  (included *vs*. dropped-out) | Test statistic | *p* |
| --- | --- | --- | --- |
| Age (2011) | 42.14 vs. 40.89 | *t* = 7.57 | < 0.001 |
| Sex (Female) | 56.0 % *vs*. 46.9 % | χ² = 29.18 | < 0.001 |
| Adulthood educational level |  |  | n.s |
| Early family risk score | -0.10 vs. 0.08 | *t* = 5.65 | < 0.001 |
| Daily smoking status | 14.1 % vs. 20.2 % | χ² = 9.39 | < 0.01 |
| Alcohol consumption |  |  | n.s. |
| Physical activity |  |  | n.s. |
| BMI | 25.98 vs. 25.62 | *t* = 2.07 | < 0.05 |
| PRS for schizophrenia | -0.06 vs. 0.09 | *t* = 3.67 | < 0.001 |
| PRS for major depression |  |  | n.s. |
| Total score of early resilience | 0.06 vs. -0.05 | *t* = 3.25 | < 0.01 |
| Index of psychological strength |  |  | n.s. |
| Index of social satisfaction | 0.04 vs. -0.04 | *t* = 1.97 | < 0.05 |
| Index of leisure time activities |  |  | n.s. |
| Index of responsible health behavior | 0.04 vs. -0.03 | *t* = 2.14 | < 0.05 |
| Index of school career | 0.06 vs. -0.05 | *t* = 2.94 | < 0.01 |
| AgeDev_Horvath_ |  |  | n.s. |
| AgeDev_Hannum_ |  |  | n.s. |
| AgeDev_Pheno_ |  |  | n.s. |
| AgeDev_Grim_ | -0.07 vs 1.01 | *t* = 3.05 | < 0.01 |
| DunedinPACE |  |  | n.s. |
|  | | | |

**Supplementary Table 4.** Results of regression analyses of Models 2 (adjusted also for early family environment and polygenic risk scores for schizophrenia and major depression). Note: Purple color indicates a change from significance to non-significance from Model 1 to Model 2. Note: An asterisk indicates statistical significance after FDR correction for multiple testing.

|  | **Index of psychological strength**  (n = 863) | | |  | **Index of social satisfaction**  (n = 1164) | | |  | **Index of leisure time activities**  (n = 1257) | | |  | **Index of responsible health behavior**  (n = 1334) | | |  | **Index of school career**  (n = 1210) | | |
| --- | --- | --- | --- | --- | --- | --- | --- | --- | --- | --- | --- | --- | --- | --- | --- | --- | --- | --- | --- |
|  | **B** | **SE** | **p** |  | **B** | **SE** | **p** |  | **B** | **SE** | **p** |  | **B** | **SE** | **p** |  | **B** | **SE** | **p** |
| **AgeDev_Horvath_** |  |  |  |  |  |  |  |  |  |  |  |  |  |  |  |  |  |  |  |
| Index | -0.184 | 0.149 | 0.216 |  | 0.010 | 0.133 | 0.943 |  | -0.146 | 0.124 | 0.237 |  | -0.098 | 0.125 | 0.436 |  | -0.247 | 0.129 | 0.055 |
| **AgeDev_Hannum_** |  |  |  |  |  |  |  |  |  |  |  |  |  |  |  |  |  |  |  |
| Index | -0.003 | 0.147 | 0.985 |  | -0.047 | 0.132 | 0.722 |  | 0.015 | 0.123 | 0.906 |  | 0.047 | 0.142 | 0.743 |  | -0.161 | 0.132 | 0.221 |
| Index^2^ | n.s. |  |  |  | n.s. |  |  |  | n.s. |  |  |  | **0.230** | **0.111** | **0.039** |  | n.s. |  |  |
| Index^3^ | n.s. |  |  |  | n.s. |  |  |  | n.s. |  |  |  | **0.032** | **0.015** | **0.034** |  | n.s. |  |  |
| **AgeDev_Pheno_** |  |  |  |  |  |  |  |  |  |  |  |  |  |  |  |  |  |  |  |
| Index | -0.242 | 0.194 | 0.213 |  | 0.057 | 0.172 | 0.739 |  | -0.234 | 0.159 | 0.140 |  | -0.188 | 0.162 | 0.247 |  | **-0.398** | **0.169** | **0.019** |
| **AgeDev_Grim_** |  |  |  |  |  |  |  |  |  |  |  |  |  |  |  |  |  |  |  |
| Index | -0.103 | 0.103 | 0.315 |  | **-0.405** | **0.139** | **0.004*** |  | -0.133 | 0.083 | 0.109 |  | **-0.595** | **0.094** | **3.78e-10*** |  | **-0.403** | **0.088** | **5.78e-06*** |
| Index^2^ | n.s. |  |  |  | **0.210** | **0.099** | **0.034** |  | n.s. |  |  |  | 0.092 | 0.074 | 0.210 |  | n.s. |  |  |
| Index^3^ | n.s. |  |  |  | **0.153** | **0.049** | **0.002*** |  | n.s. |  |  |  | **0.022** | **0.010** | **0.027** |  | n.s. |  |  |
| **DunedinPACE** |  |  |  |  |  |  |  |  |  |  |  |  |  |  |  |  |  |  |  |
| Index | 0.003 | 0.004 | 0.397 |  | 0.000 | 0.003 | 0.877 |  | **-0.009** | **0.003** | **0.001*** |  | -0.005 | 0.003 | 0.096 |  | **-0.012** | **0.003** | **0.000089*** |
| Index^2^ | n.s. |  |  |  | n.s. |  |  |  | n.s. |  |  |  | **0.008** | **0.003** | **0.003*** |  | n.s. |  |  |
| Index^3^ | n.s. |  |  |  | n.s. |  |  |  | n.s. |  |  |  | **0.001** | **0.000** | **0.004*** |  | n.s. |  |  |
| Adjusted for sex, smoking status, array type, early family environment, and genetic risk scores for schizophrenia and major depression. Note: Index^2^ and Index^3^ refer to the quadratic or cubed effect of the index, respectively. We added Index^2^ and/or Index^3^ to each model in case they were statistically significant. | | | | | | | | | | | | | | | | | | | |

**Supplementary Table 5.** Results of regression analyses of Models 3 (adjusted also for adulthood education and health behaviors). Note: Purple color indicates a change from significance to non-significance from Model 3 to Model 2. Note: An asterisk indicates statistical significance after FDR correction for multiple testing.

|  | **Index of psychological strength**  (n = 855) | | |  | **Index of social satisfaction**  (n = 1148) | | |  | **Index of leisure time activities**  (n = 1239) | | |  | **Index of responsible health behavior**  (n = 1314) | | |  | **Index of school career**  (n = 1197) | | |
| --- | --- | --- | --- | --- | --- | --- | --- | --- | --- | --- | --- | --- | --- | --- | --- | --- | --- | --- | --- |
|  | **B** | **SE** | **p** |  | **B** | **SE** | **p** |  | **B** | **SE** | **p** |  | **B** | **SE** | **p** |  | **B** | **SE** | **p** |
| **AgeDev_Horvath_** |  |  |  |  |  |  |  |  |  |  |  |  |  |  |  |  |  |  |  |
| Index | -0.195 | 0.151 | 0.197 |  | -0.025 | 0.135 | 0.851 |  | -0.177 | 0.133 | 0.184 |  | -0.100 | 0.131 | 0.446 |  | -0.234 | 0.135 | 0.082 |
| **AgeDev_Hannum_** |  |  |  |  |  |  |  |  |  |  |  |  |  |  |  |  |  |  |  |
| Index | 0.015 | 0.149 | 0.921 |  | -0.039 | 0.134 | 0.769 |  | 0.061 | 0.133 | 0.644 |  | 0.163 | 0.150 | 0.277 |  | -0.109 | 0.138 | 0.428 |
| Index^2^ | n.s. |  |  |  | n.s. |  |  |  | n.s. |  |  |  | 0.258 | **0.112** | **0.022** |  | n.s. |  |  |
| Index^3^ | n.s. |  |  |  | n.s. |  |  |  | n.s. |  |  |  | 0.034 | **0.015** | **0.027** |  | n.s. |  |  |
| **AgeDev_Pheno_** |  |  |  |  |  |  |  |  |  |  |  |  |  |  |  |  |  |  |  |
| Index | -0.220 | 0.196 | 0.260 |  | 0.069 | 0.172 | 0.689 |  | -0.074 | 0.168 | 0.660 |  | -0.007 | 0.167 | 0.967 |  | -0.190 | 0.174 | 0.275 |
| **AgeDev_Grim_** |  |  |  |  |  |  |  |  |  |  |  |  |  |  |  |  |  |  |  |
| Index | -0.066 | 0.100 | 0.510 |  | **-0.316** | **0.136** | **0.021*** |  | -0.008 | 0.086 | 0.926 |  | **-0.391** | **0.085** | **4.44e-06*** |  | **-0.281** | **0.090** | **0.002*** |
| Index^2^ | n.s. |  |  |  | **0.193** | **0.096** | **0.045** |  | n.s. |  |  |  | n.s. |  |  |  | n.s. |  |  |
| Index^3^ | n.s. |  |  |  | **0.136** | **0.049** | **0.005*** |  | n.s. |  |  |  | n.s. |  |  |  | n.s. |  |  |
| **DunedinPACE** |  |  |  |  |  |  |  |  |  |  |  |  |  |  |  |  |  |  |  |
| Index | 0.004 | 0.003 | 0.206 |  | 0.001 | 0.003 | 0.607 |  | -0.004 | 0.003 | 0.125 |  | -0.003 | 0.003 | 0.368 |  | **-0.006** | **0.003** | **0.047** |
| Index^2^ | n.s. |  |  |  | n.s. |  |  |  | n.s. |  |  |  | **0.005** | **0.002** | **0.025** |  | n.s. |  |  |
| Index^3^ | n.s. |  |  |  | n.s. |  |  |  | n.s. |  |  |  | **0.001** | **0.000** | **0.020*** |  | n.s. |  |  |
| Adjusted for sex, array type, smoking status, early family environment, polygenic risk for schizophrenia and major depression, adulthood education, and adulthood health behaviors (alcohol consumption, physical activity, body-mass index). Note: Index^2^ and Index^3^ refer to the quadratic or cubed effect of the index, respectively. We added Index^2^ and/or Index^3^ to each model in case they were statistically significant. | | | | | | | | | | | | | | | | | | | |

**Supplementary Table 6.** Results of regression analyses when predicting epigenetic ageing and only including EPIC array data. Note: Green color indicates a new association that emerged significant in these sensitivity analyses. Note: An asterisk indicates statistical significance after FDR correction for multiple testing.

|  | **Index of psychological strength**  (n = 823) | | |  | **Index of social satisfaction**  (n = 1177) | | |  | **Index of leisure time activities**  (n = 1276) | | |  | **Index of responsible health behavior**  (n = 1371) | | |  | **Index of school career**  (n = 1229) | | |
| --- | --- | --- | --- | --- | --- | --- | --- | --- | --- | --- | --- | --- | --- | --- | --- | --- | --- | --- | --- |
|  | **B** | **SE** | **p** |  | **B** | **SE** | **p** |  | **B** | **SE** | **p** |  | **B** | **SE** | **p** |  | **B** | **SE** | **p** |
| **AgeDev_Horvath_** |  |  |  |  |  |  |  |  |  |  |  |  |  |  |  |  |  |  |  |
| Index | -0.267 | 0.147 | 0.068 |  | -0.043 | 0.130 | 0.738 |  | -0.105 | 0.121 | 0.383 |  | -0.166 | 0.121 | 0.169 |  | **-0.246** | **0.122** | **0.044** |
| **AgeDev_Hannum_** |  |  |  |  |  |  |  |  |  |  |  |  |  |  |  |  |  |  |  |
| Index | -0.074 | 0.147 | 0.613 |  | -0.010 | 0.130 | 0.940 |  | 0.389 | 0.207 | 0.060 |  | 0.032 | 0.140 | 0.817 |  | -0.129 | 0.125 | 0.301 |
| Index^2^ | n.s. |  |  |  | n.s. |  |  |  | -0.076 | 0.093 | 0.412 |  | **0.209** | **0.105** | **0.047** |  | n.s. |  |  |
| Index^3^ | n.s. |  |  |  | n.s. |  |  |  | **-0.130** | **0.064** | **0.042** |  | **0.030** | **0.015** | **0.038** |  | n.s. |  |  |
| **AgeDev_Pheno_** |  |  |  |  |  |  |  |  |  |  |  |  |  |  |  |  |  |  |  |
| Index | **-0.437** | **0.189** | **0.021** |  | 0.011 | 0.165 | 0.946 |  | -0.231 | 0.152 | 0.129 |  | -0.256 | 0.153 | 0.095 |  | **-0.449** | **0.156** | **0.004*** |
| **AgeDev_Grim_** |  |  |  |  |  |  |  |  |  |  |  |  |  |  |  |  |  |  |  |
| Index | **-0.244** | **0.106** | **0.021** |  | **-0.429** | **0.142** | **0.003*** |  | -0.154 | 0.084 | 0.066 |  | **-0.712** | **0.095** | **1.042e-13*** |  | **-0.458** | **0.086** | **1.117e-07*** |
| Index^2^ | n.s. |  |  |  | **0.258** | **0.102** | **0.011** |  | n.s. |  |  |  | **-0.070** | **0.034** | **0.043** |  | n.s. |  |  |
| Index^3^ | n.s. |  |  |  | **0.169** | **0.051** | **0.001*** |  | n.s. |  |  |  | n.s. |  |  |  | n.s. |  |  |
| **DunedinPACE** |  |  |  |  |  |  |  |  |  |  |  |  |  |  |  |  |  |  |  |
| Index | -0.003 | 0.004 | 0.326 |  | 0.000 | 0.003 | 0.890 |  | **-0.009** | **0.003** | **0.002*** |  | **-0.008** | **0.003** | **0.009*** |  | **-0.016** | **0.003** | **1.947e-08*** |
| Index^2^ | n.s. |  |  |  | n.s. |  |  |  | n.s. |  |  |  | **0.006** | **0.002** | **0.013*** |  | n.s. |  |  |
| Index^3^ | n.s. |  |  |  | n.s. |  |  |  | n.s. |  |  |  | **0.001** | **0.000** | **0.010*** |  | n.s. |  |  |
| Adjusted for sex, array type, and smoking status. Note: Index^2^ and Index^3^ refer to the quadratic or cubed effect of the index, respectively. We added Index^2^ and/or Index^3^ to each model in case they were statistically significant. | | | | | | | | | | | | | | | | | | | |

**Supplementary Table 7.** Results of regression analyses when controlling for cell types. Note: An asterisk indicates statistical significance after FDR correction for multiple testing.

|  | **Index of psychological strength**  (n = 990) | |  | **Index of social satisfaction**  (n = 1333) | | |  | **Index of leisure time activities**  (n = 1455) | | | |  | | **Index of responsible health behavior**  (n = 1551) | | |  | | **Index of school career**  (n = 1394) | | |  | | **Total score of early resilience**  (n = 1593) | | |  |
| --- | --- | --- | --- | --- | --- | --- | --- | --- | --- | --- | --- | --- | --- | --- | --- | --- | --- | --- | --- | --- | --- | --- | --- | --- | --- | --- | --- |
|  | **B** | **p** | |  | **B** | **p** | | |  | **B** | **p** | |  | | **B** | **p** | |  | | **B** | **p** | |  | | **B** | **p** | |
| **AgeDev_Horvath_** |  |  | |  |  |  | | |  |  |  | |  | |  |  | |  | |  |  | |  | |  |  | |
| Index | -0.160 | 0.225 | |  | 0.004 | 0.977 | | |  | -0.138 | 0.213 | |  | | -0.181 | 0.111 | |  | | **-0.261** | **0.021** | |  | | **-0.232** | **0.048** | |
| Index^2^ | n.s. |  | |  | n.s. |  | | |  | n.s. |  | |  | | n.s. |  | |  | | n.s. |  | |  | | n.s. |  | |
| Index^3^ | n.s. |  | |  | n.s. |  | | |  | n.s. |  | |  | | n.s. |  | |  | | n.s. |  | |  | | n.s. |  | |
| **AgeDev_Hannum_** |  |  | |  |  |  | | |  |  |  | |  | |  |  | |  | |  |  | |  | |  |  | |
| Index | -0.018 | 0.879 | |  | -0.010 | 0.924 | | |  | **0.456** | **0.007*** | |  | | -0.055 | 0.592 | |  | | -0.072 | 0.493 | |  | | -0.048 | 0.650 | |
| Index^2^ | n.s. |  | |  | n.s. |  | | |  | -0.051 | 0.499 | |  | | n.s. |  | |  | | n.s. |  | |  | | n.s. |  | |
| Index^3^ | n.s. |  | |  | n.s. |  | | |  | **-0.130** | **0.013*** | |  | | n.s. |  | |  | | n.s. |  | |  | | n.s. |  | |
| **AgeDev_Pheno_** |  |  | |  |  |  | | |  |  |  | |  | |  |  | |  | |  |  | |  | |  |  | |
| Index | **-0.346** | **0.027** | |  | 0.073 | 0.611 | | |  | -0.222 | 0.087 | |  | | **-0.368** | **0.006*** | |  | | **-0.347** | **0.010***  **0.961** | |  | | **-0.420** | **0.002*** | |
| Index^2^ | n.s. |  | |  | n.s. |  | | |  | n.s. |  | |  | | n.s. |  | |  | | n.s. |  | |  | | n.s. |  | |
| Index^3^ | n.s. |  | |  | n.s. |  | | |  | n.s. |  | |  | | n.s. |  | |  | | n.s. |  | |  | | n.s. |  | |
| **AgeDev_Grim_** |  |  | |  |  |  | | |  |  |  | |  | |  |  | |  | |  |  | |  | |  |  | |
| Index | **-0.201** | **0.025** | |  | **-0.377** | **0.003*** | | |  | -0.142 | 0.054 | |  | | **-0.650** | **3.45e-18*** | |  | | **-0.375** | **9.61e-07*** | |  | | **-0.540** | **5.89e-12*** | |
| Index^2^ | n.s. |  | |  | **0.206** | **0.024** | | |  | n.s. |  | |  | | n.s. |  | |  | | n.s. |  | |  | | n.s. |  | |
| Index^3^ | n.s. |  | |  | **0.134** | **0.004*** | | |  | n.s. |  | |  | | n.s. |  | |  | | n.s. |  | |  | | n.s. |  | |
| **DunedinPACE** |  |  | |  |  |  | | |  |  |  | |  | |  |  | |  | |  |  | |  | |  |  | |
| Index | -0.002 | 0.414 | |  | 0.000 | 0.918 | | |  | **-0.009** | **0.00024*** | |  | | **-0.009** | **0.003*** | |  | | **-0.014** | **4.29e-08*** | |  | | **-0.014** | **4.19e-08*** | |
| Index^2^ | n.s. |  | |  | n.s. |  | | |  | n.s. |  | |  | | **0.007** | **0.003*** | |  | | n.s. |  | |  | | n.s. |  | |
| Index^3^ | n.s. |  | |  | n.s. |  | | |  | n.s. |  | |  | | **0.001** | **0.005*** | |  | | n.s. |  | |  | | n.s. |  | |
| Adjusted for sex, smoking status, array type, and cell types (CD8+ T cells, CD4+ T cells, NK cells, B cells, and monocytes). To avoid the risk of over-fitting, we excluded one cell type from the covariates (i.e., granulocytes). Note: Index^2^ and Index^3^ refer to the quadratic or cubed effect of the index, respectively. We added Index^2^ and/or Index^3^ to each model in case they were statistically significant. | | | | | | | | | | | | | | | | | | | | | | | | | | |  |

**Supplementary Table 8.** Results of regression analyses when predicting additional variables of epigenetic ageing. Note: Green color indicates a new association that emerged significant in these sensitivity analyses. Purple color, in turn, indicates an association that turned non-significant in these sensitivity analyses. Note: An asterisk indicates statistical significance after FDR correction for multiple testing.

|  | **Index of psychological strength**  (n = 990) | | |  | **Index of social satisfaction**  (n = 1333) | | |  | **Index of leisure time activities**  (n = 1455) | | |  | **Index of responsible health behavior**  (n = 1551) | | |  | **Index of school career**  (n = 1394) | | |
| --- | --- | --- | --- | --- | --- | --- | --- | --- | --- | --- | --- | --- | --- | --- | --- | --- | --- | --- | --- |
|  | **B** | **SE** | **p** |  | **B** | **SE** | **p** |  | **B** | **SE** | **p** |  | **B** | **SE** | **p** |  | **B** | **SE** | **p** |
| **IEAA_Horvath_** |  |  |  |  |  |  |  |  |  |  |  |  |  |  |  |  |  |  |  |
| Index | -0.195 | 0.132 | 0.140 |  | -0.047 | 0.120 | 0.695 |  | -0.113 | 0.110 | 0.303 |  | -0.188 | 0.112 | 0.095 |  | **-0.332** | **0.113** | **0.003*** |
| **AgeDevPC_Horvath_** |  |  |  |  |  |  |  |  |  |  |  |  |  |  |  |  |  |  |  |
| Index | -0.070 | 0.107 | 0.512 |  | -0.064 | 0.097 | 0.507 |  | -0.141 | 0.088 | 0.111 |  | 0.020 | 0.091 | 0.828 |  | **-0.223** | **0.092** | **0.015*** |
| **IEAA_Hannum_** |  |  |  |  |  |  |  |  |  |  |  |  |  |  |  |  |  |  |  |
| Index | -0.029 | 0.122 | 0.811 |  | 0.029 | 0.111 | 0.791 |  | **0.470** | **0.171** | **0.006*** |  | -0.009 | 0.104 | 0.934 |  | -0.140 | 0.104 | 0.182 |
| Index^2^ | n.s. |  |  |  | n.s. |  |  |  | -0.036 | 0.077 | 0.645 |  | n.s. |  |  |  | n.s. |  |  |
| Index^3^ | n.s. |  |  |  | n.s. |  |  |  | **-0.130** | **0.052** | **0.013*** |  | n.s. |  |  |  | n.s. |  |  |
| **EEAA_Hannum_** |  |  |  |  |  |  |  |  |  |  |  |  |  |  |  |  |  |  |  |
| Index | 0.0334 | 0.162 | 0.832 |  | -0.061 | 0.148 | 0.682 |  | **0.484** | **0.230** | **0.035** |  | 0.082 | 0.158 | 0.604 |  | -0.116 | 0.142 | 0.413 |
| Index^2^ | n.s. |  |  |  | n.s. |  |  |  | -0.090 | 0.104 | 0.385 |  | **0.250** | **0.121** | **0.040** |  | n.s. |  |  |
| Index^3^ | n.s. |  |  |  | n.s. |  |  |  | **-0.152** | **0.070** | **0.031** |  | **0.034** | **0.017** | **0.046** |  | n.s. |  |  |
| **AgeDevPC_Hannum_** |  |  |  |  |  |  |  |  |  |  |  |  |  |  |  |  |  |  |  |
| Index | 0.035 | 0.105 | 0.739 |  | -0.027 | 0.093 | 0.772 |  | -0.039 | 0.086 | 0.645 |  | -0.021 | 0.088 | 0.811 |  | -0.131 | 0.090 | 0.146 |
| **AgeDevPC_Pheno_** |  |  |  |  |  |  |  |  |  |  |  |  |  |  |  |  |  |  |  |
| Index | -0.044 | 0.145 | 0.762 |  | **-0.391** | **0.201** | **0.052** |  | -0.226 | 0.117 | 0.053 |  | -0.084 | 0.120 | 0.482 |  | **-0.368** | **0.122** | **0.003*** |
| Index^2^ | n.s. |  |  |  | **0.309** | **0.144** | **0.032** |  | n.s. |  |  |  | n.s. |  |  |  | n.s. |  |  |
| Index^3^ | n.s. |  |  |  | **0.147** | **0.073** | **0.044** |  | n.s. |  |  |  | n.s. |  |  |  | n.s. |  |  |
| **AgeDevPC_Grim_** |  |  |  |  |  |  |  |  |  |  |  |  |  |  |  |  |  |  |  |
| Index | **-0.333** | **0.123** | **0.007*** |  | -0.073 | 0.069 | 0.296 |  | **-0.130** | **0.064** | **0.041** |  | **-0.519** | **0.064** | **1.096e-15*** |  | **-0.364** | **0.067** | **5.818e-08*** |
| Index^2^ | 0.106 | 0.071 | 0.135 |  | n.s. |  |  |  | n.s. |  |  |  | n.s. |  |  |  | n.s. |  |  |
| Index^3^ | **0.101** | **0.035** | **0.004*** |  | n.s. |  |  |  | n.s. |  |  |  | n.s. |  |  |  | n.s. |  |  |
| Adjusted for sex, array type, and smoking status. Note: Index^2^ and Index^3^ refer to the quadratic or cubed effect of the index, respectively. We added Index^2^ and/or Index^3^ to each model in case they were statistically significant. | | | | | | | | | | | | | | | | | | | |

**Supplementary Table 9.** Results of regression analyses when predicting each epigenetic clock in 2011 by the interaction between total score of early resilience and each epigenetic clock in 1986. Note: An asterisk indicates statistical significance after FDR correction for multiple testing.

|  | **B** | **SE** | **p** |
| --- | --- | --- | --- |
| **AgeDev_Horvath_ in 2011** |  |  |  |
| Total early resilience | -0.009 | 0.072 | 0.902 |
| AgeDev_Horvath_ in 1986 | **0.473** | **0.056** | **1.071e-15*** |
| Interaction | -0.020 | 0.062 | 0.741 |
| **AgeDev_Hannum_ in 2011** |  |  |  |
| Total early resilience | 0.038 | 0.068 | 0.578 |
| AgeDev_Hannum_ in 1986 | **0.360** | **0.053** | **5.727e-11*** |
| Interaction | -0.075 | 0.057 | 0.188 |
| **AgeDev_Pheno_ in 2011** |  |  |  |
| Total early resilience | 0.033 | 0.073 | 0.654 |
| AgeDev_Pheno_ in 1986 | **0.440** | **0.056** | **1.291e-13*** |
| Interaction | -0.088 | 0.057 | 0.125 |
| **AgeDev_Grim_ in 2011** |  |  |  |
| Total early resilience | 0.016 | 0.050 | 0.757 |
| AgeDev_Grim_ in 1986 | **0.380** | **0.042** | **2.327e-17*** |
| Interaction | **-0.081** | **0.041** | **0.047** |
| **DunedinPACE in 2011** |  |  |  |
| Total early resilience | 0.021 | 0.065 | 0.743 |
| DunedinPACE in 1986 | **0.545** | **0.050** | **1.431e-23*** |
| Interaction | 0.023 | 0.054 | 0.670 |
| Adjusted for sex, array type in 1986 and 2011, and smoking status in 2011 and 1986. The clock variables were standardized before adding to the model (mean = 0, SD = 1). | | | |
